# Supplementary material for: Patient-oriented risk score for predicting death 1 year after myocardial infarction: the SweDen risk score
Source: Open Heart. 2022 Dec 1;9(2):e002143. doi: 10.1136/openhrt-2022-002143 (PMC9723953; doi:10.1136/openhrt-2022-002143)
Supplement: Supplementary data [file openhrt-2022-002143supp001.pdf]

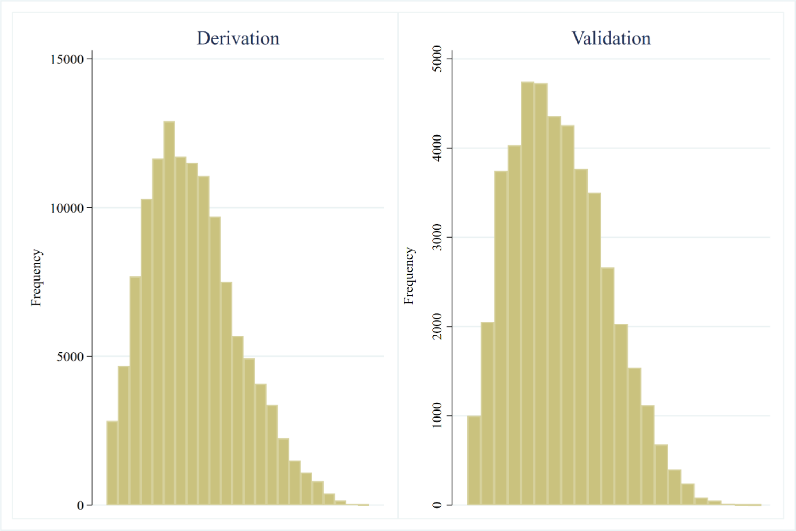

Figure 3. Histograms of the linear predictor

Table 3.

Measure	Derivation data		Validation data	
	Estimate	se	Estimate	Se
HR; Fairly good vs. good*	2.78	.217	2.55	.376
HR; Fairly bad vs. good*	10.45	.772	7.47	1.047
HR; Bad vs. good*	33.69	2.466	21.27	2.965

HR; hazard ratios

Calculation of risk score

Step 1. Cox proportional hazards regression log hazard coefficients

Risk factor	Coefficient, B_i	Mean or proportion
Age, years	.07801	73.3
Gender	.08949	.64
Diabetes	.51539	.22
Smoker previous	.03841	.35
Smoker current	.38103	.17
Smoker non	Base	.48
Statins not prescribed	.65660	.13
Heart failure	.96994	.14

Step 2. Definition of a point

One point constitutes:

5 years aging multiplied by the coefficient for Age

From our model, we have B_i = .07801*5= 0.39005

3. Formula for points

Categories		Reference value(W_{ij})	B_i	Pointsij=$B_i(W_{ij}-W_iREF)/B$
			.07801	
Age	55-59	57= W_1REF		0
	60-64	62		1
	65-69	67		2
	70-74	72		3
	75-79	77		4
	80-84	82		5
	85-89	87		6
	90-94	92		7
	95-105	97		8
Gender	Female	0=W_2REF	.08949	0
	Male	1		0
Diabetes	No	0=W_3REF	.51539	0
	Yes	1		1
Smoker previous	No	0=W_4REF	.03841	0
	Yes	1		0
Smoker current	No	0=W_4REF	.38103	0
	Yes	1		1
Statins	Prescribed	0=W_5REF	.65660	0
	Not prescribed	1		2
Heart failure	No	0=W_6REF	.96994	0
	Yes	1		2

4. Point System

Risk factor	Categories	Points
Age	55-59	0
	60-64	1
	65-69	2
	70-74	3
	75-79	4
	80-84	5
	85-89	6
	90-94	7
	95-105	8
Gender	Female	0
	Male	0
Diabetes	No	0
	Yes	1
Smoker previous	No	0
	Yes	0
Smoker current	No	0
	Yes	1
Statins	Prescribed	0
	Not prescribed	2
Heart failure	No	0
	Yes	2

5. Attach risk to every point

$\sum_{i=1}^{p} B_i\overline{X}_i =$ .0780096*(73.3) + .0894894*(0.64) + .5153974*(0.23) + .0384073*(0.35) + .3810368*(0.17) +.6566036*(0.13) + .9699417*(0.14) = 6.1932874

$\sum_{i=1}^{p} B_i X_i \approx$ for every point

.0780096*(57)+.390048*(0) = 4.4465472

.0780096*(57)+.390048*(1) = 4.8365952

.0780096*(57)+.390048*(2) = 5.2266432

.0780096*(57)+.390048*(3) = 5.6166912

.0780096*(57)+.390048*(4) = 6.0067392

.0780096*(57)+.390048*(5) = 6.3967872

.0780096*(57)+.390048*(6) = 6.7868352

.0780096*(57)+.390048*(7) = 7.1768832

.0780096*(57)+.390048*(8) = 7.5669312

.0780096*(57)+.390048*(9) = 7.9569792

.0780096*(57)+.390048*(10) = 8.3470272

.0780096*(57)+.390048*(11) = 8.7370752

.0780096*(57)+.390048*(12) = 9.1271232

.0780096*(57)+.390048*(13) = 9.5171712

.0780096*(57)+.390048*(14) = 9.9072192

$S_o(t)$= 0.9035 Survival at one-year

5) Attach risk estimate to each point total risk=$1 - S_o(t)^{exp\left(\sum_{i=1}^{p} B_i X_i - \sum_{i=1}^{p} B_i \overline{X}_i\right)}$

Points	Risks associated with point totals
0	0.0176
1	0.0259
2	0.0380
3	0.0557
4	0.0812
5	0.1176
6	0.1687
7	0.2389
8	0.3318
9	0.4488
10	0.5851
11	0.7273
12	0.8533
13	0.9412
14	0.9848
